# Disseminated Blastomycosis

**DOI:** 10.4269/ajtmh.24-0133

**Published:** 2024-07-23

**Authors:** Ashton D. Hall, Kavya Patel, Michael B. Burch, Lea M. Rotert

**Affiliations:** ^1^ Division of Infectious Diseases, Department of Internal Medicine, University of Cincinnati College of Medicine, Cincinnati, Ohio;; ^2^ Department of Radiology, University of Cincinnati College of Medicine, Cincinnati, Ohio

**Figure 1.**
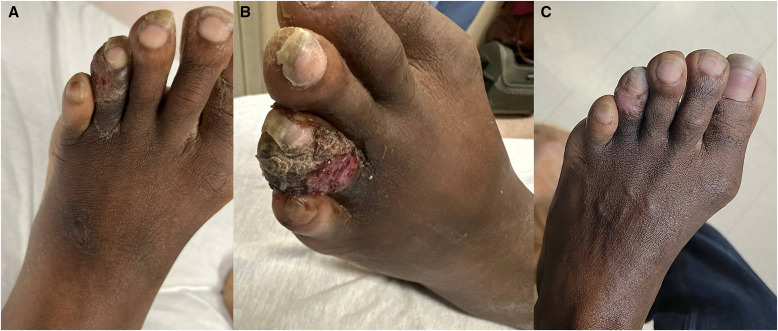
Fourth toe of the left foot at initial presentation (**A**), readmission (**B**), and outpatient follow-up at 3 months (**C**). Progressively worsening ulceration of the dorsal aspect with irregularly raised hyperkeratotic tissue, serous drainage, and eventual scarring.

**Figure 2.**
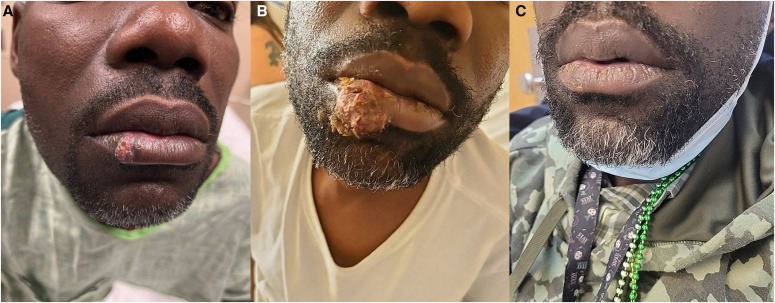
Elevated ulcerative lesion of the right lower lip at initial presentation (**A**), readmission (**B**), and outpatient follow up at 3 months (**C**) with minimal scarring.

**Figure 3.**
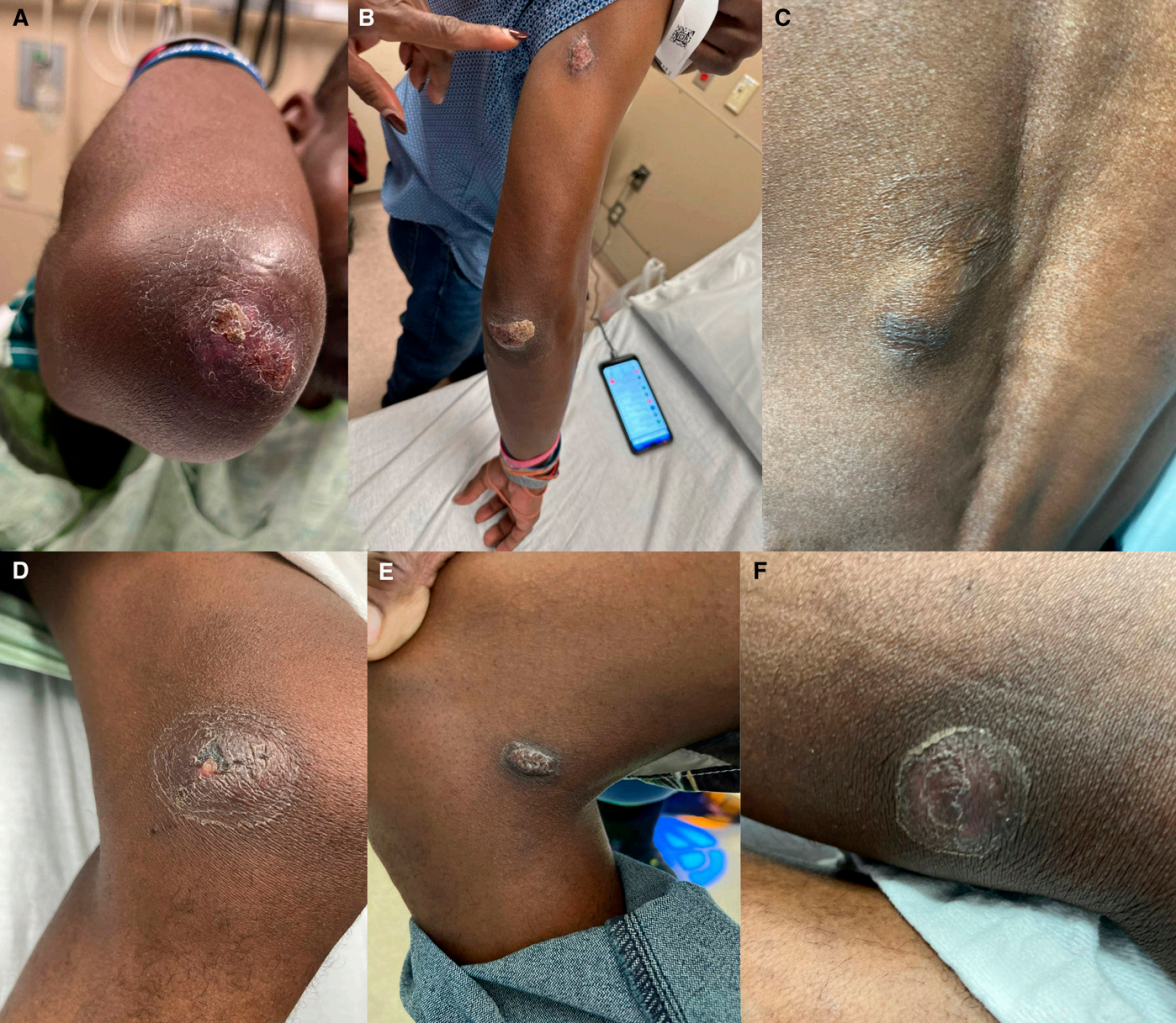
Psoriasiform plaque of the right elbow at initial presentation (**A**) and readmission (**B**). An assortment of brown to pink, scaly and verrucous, slightly fluctuant hyperkeratotic plaques of the left midback (**C**), left and right inner leg (**D** and **E**), and left calf (**F**) on initial presentation. Follow-up images were not available.

A 41-year-old man presented with a 1-month history of suppurative skin lesions, dry cough, and unintentional weight loss of 25 pounds. Lesions first appeared on the left fourth toe and subsequently involved the calf, abdomen, back, elbow, chin, and lower lip (Figures 1–3). The patient presented to our emergency department after doxycycline, trimethoprim-sulfamethoxazole, and mupirocin did not lead to relief. The patient has lived in Cincinnati for the last 25 years. He worked as a corporate housekeeper and denied recent travel, traumatic injury, pet ownership, and exposure to the outdoors or animals.

**Figure 4.**
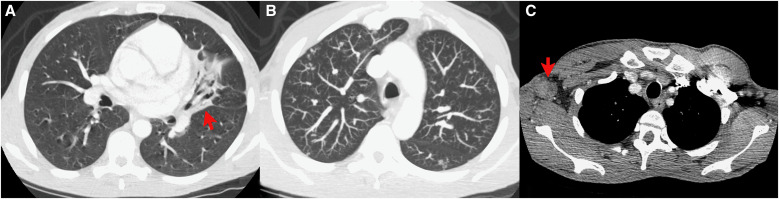
(**A**) Computed tomography chest scan on readmission showing focal scarring with bronchiectasis in the inferior left upper lobe. (**B**) Maximum intensity projection image showing multiple scattered lung nodules in both upper lobes. (**C**) Right axillary lymphadenopathy measuring 3.2 × 2.3 cm with possible necrosis. Courtesy of M. B. Burch at the University of Cincinnati.

**Figure 5.**
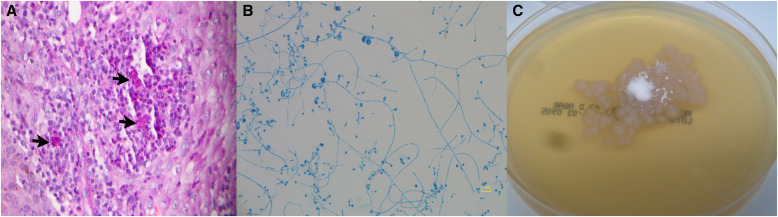
(**A**) Skin biopsy specimen with periodic acid-Schiff stain with diastase at 40× magnification showing broad-based budding yeasts of 10 *μ*m surrounded by neutrophils in an epidermal microabscess. Courtesy of K. E. Spicknall at the University of Cincinnati. (**B**) Microscopic image of *Blastomyces dermatitidis* stained with lactophenol aniline blue showing the characteristic round or oval single conidia at the apex of a conidiophore. (**C**) *Blastomyces dermatitidis* growing on inhibitory mold agar at 25°C. Courtesy of N. L. Wengenack and the Mayo Clinic Mycology Laboratory, where the patient’s sample was processed.

He was seen 3 weeks later with enlarging skin lesions and denied systemic symptoms. Examination revealed rhonchi and palpable cervical lymph nodes bilaterally. A computed tomography scan of the chest was notable for pulmonary nodules without cavitary lesions, bronchiectactic scarring, and bilateral axillary lymphadenopathy (Figure 4). He was started on azithromycin and ceftriaxone for suspected community-acquired pneumonia. Testing was negative for HIV, Mpox, tuberculosis, syphilis, and *Cryptococcus neoformans*. Blood and sputum cultures were negative. A punch biopsy specimen of a right arm lesion showed 10-*µ*m yeast forms by periodic acid-Schiff staining with diastase (Figure 5). Notably, the yeast forms of *Histoplasma capsulatum* are ∼4 *µ*m within macrophages. Grocott-Gomori methenamine silver (GMS) staining was not performed. Serum antigen studies were negative for *Blastomyces* (0.59 ng/mL) and *Aspergillus* (optical density index, 0.04; Fungitell assay result, <31 pg/mL) but positive for *Histoplasma* (1.41 ng/mL). Urine antigen studies were positive for *Blastomyces* (1.48 ng/mL) and *Histoplasma* (2.98 ng/mL). The *Blastomyces* serology titer was 1:2; *Histoplasma* serology could not be determined because of anticomplementary activity. Tissue samples from the right arm biopsy specimen grew *Blastomyces* spp. 2 months later (Figure 5). Liposomal amphotericin was started at 3 mg/kg/day, which was changed to itraconazole 300 mg twice daily 4 days later upon demonstration of blastomycosis. On 3-month follow-up, all skin lesions had healed (Figures 1 and 2). Itraconazole was discontinued after 6 months after resolution of all signs and symptoms of infection.

Blastomycosis, caused by the thermally dimorphic molds *Blastomyces dermatitidis* and *Blastomyces gilchristii*, is historically endemic in the Great Lakes-St. Lawrence Seaway region, the Ohio and Mississippi River Valleys, the southeastern United States, and several Canadian provinces.^1^ From 2019 to 2021, 719 blastomycosis cases were reported in the United States, with most requiring hospitalization.^2^ Recent reports indicate an underestimated incidence and geographic distribution of blastomycosis across the United States, particularly in Vermont.^3^

Diagnosis relies on the physician’s high index of clinical suspicion, such as recognizing blastomycosis as a potential diagnosis in places of endemicity with a compatible lung or disseminated illness.^4^^,^^5^ Risk factors for blastomycosis include current or former residence in or travel to areas of high endemicity; outdoor activities with exposure to decaying organic matter, such as hunting, camping, and hiking; and dog ownership, particularly if the animal has been recently diagnosed with pneumonia.^4^^,^^5^ Antigen detection assays, performed on urine, serum, cerebrospinal fluid, and bronchoalveolar lavage fluid, may exhibit cross-reactivity with histoplasmosis (up to 90% in urine) and less commonly with paracoccidioidomycosis or talaromycosis.^4^^,^^5^ Histopathology, best performed with GMS staining, often makes a presumptive diagnosis when 8- to 15-*µ*m, thick, broad-based budding yeasts with refractile cell walls are seen.^4^^,^^6^ All patients with blastomycosis are treated with antifungals to prevent disease progression; the choice of treatment depends on clinical presentation.^4^
